# Double MgO-Based Perpendicular Magnetic Tunnel Junction for Artificial Neuron

**DOI:** 10.3389/fnins.2020.00309

**Published:** 2020-04-30

**Authors:** Dong Won Kim, Woo Seok Yi, Jin Young Choi, Kei Ashiba, Jong Ung Baek, Han Sol Jun, Jae Joon Kim, Jea Gun Park

**Affiliations:** ^1^Department of Nanoscale Semiconductor Engineering, Hanyang University, Seoul, South Korea; ^2^Department of Creative IT Engineering, Pohang University of Science and Technology, Pohang, South Korea; ^3^MRAM Center, Department of Electronics and Computer Engineering, Hanyang University, Seoul, South Korea; ^4^Wafer Engineering Department, SUMCO Corporation, Imari, Japan

**Keywords:** neuromorphic, MRAM, spiking neuron, spiking neural network, artificial neuron

## Abstract

A perpendicular spin transfer torque (p-STT)-based neuron was developed for a spiking neural network (SNN). It demonstrated the integration behavior of a typical neuron in an SNN; in particular, the integration behavior corresponding to magnetic resistance change gradually increased with the input spike number. This behavior occurred when the spin electron directions between double Co_2_Fe_6_B_2_ free and pinned layers in the p-STT-based neuron were switched from parallel to antiparallel states. In addition, a neuron circuit for integrate-and-fire operation was proposed. Finally, pattern-recognition simulation was performed for a single-layer SNN.

## Introduction

Artificial neural network (ANN)-based artificial intelligence (AI) has been one of the most successful technologies in recent years. Today, it is applied in numerous fields, such as education, security, finance, science, and entertainment. In particular, the performance of the AI has already exceeded the ability of human beings (Szegedy et al., 2015; He et al., 2016; Silver et al., 2016; Hu J. et al., 2018) in fields such as image recognition and the Go game. However, there is a limitation to conventional ANNs working on the von-Neumann architecture. The low bandwidth between processor and memory in the von-Neumann architecture hinders efficient neural networks processing (Merolla et al., 2014; Monroe, 2014). Neuromorphic computing systems that mimic the human brain has been designed to overcome this limitation using complementary metal oxide semiconductor (CMOS)-based artificial neuron devices. However, it is a major challenge to implement high neuronal density by means of conventional CMOS technology because emulating the integration function of the neuron relies on the capacitor where the area of capacitor would be prohibitively large (∼1,000 F^2^) to obtain the desired capacitance (∼10 fF/μm^2^) (Gentet et al., 2000; Indiveri et al., 2013). Therefore, an artificial neuron device without a capacitor is necessary to implement high-density neuromorphic chip. Recently, emerging artificial neuron devices have been reported as an alternative to CMOS-based neuron devices such as partially depleted silicon-on-insulator n-MOSFET (PD-SOI n-MOSFET) (Dutta et al., 2017), phase change random-access memory (PCRAM) (Tuma et al., 2016), and magnetic random-access memory (MRAM) (Grollier et al., 2016; Sengupta et al., 2016; Shim et al., 2017; Srinivasan et al., 2017; Torrejon et al., 2017; Mizrahi et al., 2018; Kurenkov et al., 2019). Among them, MRAM has been proposed as a promising candidate for artificial neuron device due to its high-area efficiency, fast operating speed, and low power consumption (Zhang et al., 2016; Liyanagedera et al., 2017; Hu G. et al., 2018). However, past researches have mainly focused on stochastic behavior of MRAM, and its integration behavior has not yet been reported. In this work, we first demonstrated the integration behavior of perpendicular spin transfer torque magnetic tunneling junction (p-STT MTJ) spin valve when switching from parallel to antiparallel states between Co_2_Fe_6_B_2_ free and pinned layers. In addition, its integration behavior was discussed with grain boundary in MgO tunneling barrier. Finally, we conducted a pattern recognition simulation of a spiking neural network (SNN) using our p-STT-based neuron.

## Materials and Methods

### Device Fabrication

#### p-STT MTJ

A p-STT MTJ spin valve structure was fabricated using a 12-in SiO_2_ wafer multichamber cluster magnetron sputtering system under a high vacuum of <1 × 10^–8^ Torr. In particular, it was vertically stacked with a W/TiN bottom electrode, Ta buffer layer, Pt seed layer, [Co (0.47 nm)/Pt (0.23 nm)]_6_/Co (0.51 nm) lower SyAF layer, Ru spacer layer (0.85 nm), Co (0.51 nm)/Pt (0.23 nm)/[Co (0.47 nm)/Pt (0.23 nm)]_3_ upper SyAF layer, Co buffer layer (0.4 nm), W bridge layer (0.2 nm), Co_2_Fe_6_B_2_ pinned layer (0.95 nm), MgO tunneling barrier (1.0 nm), Fe insertion layer (0.3 nm), Co_2_Fe_6_B_2_ lower free layer (0.8 nm), W spacer layer (0.4 nm), Co_2_Fe_6_B_2_ upper free layer (0.8 nm), MgO capping layer (0.8 nm)/Fe diffusion barrier (0.19 nm), W capping layer (4.0 nm), and Ta/Ru top electrode. An amorphous Ta buffer layer was used to prevent the texturing of the polycrystallinity of the W/TiN bottom electrode. A Pt seed layer thickness was optimized for the face-centered cubic (f.c.c) texturing of the [Co/Pt] SyAF multilayers. The [Co/Pt]_6_ lower SyAF layer and [CoPt]_3_ upper SyAF layer were perfectly antiferromagnetic coupled by inserting an optimized Ru spacer layer by Ruderman–Kittel–Kasuya–Yosida (RKKY) coupling. Then, the Co_2_Fe_6_B_2_ pinned layer was ferrocoupled to the [CoPt]_3_ upper SyAF layer by a W bridge layer. Then, the p-STT MTJ spin valve was *ex situ* annealed at 350°C for 30 min under a vacuum below 10^–6^ Torr and a perpendicular magnetic field of 3 T. The p-STT MTJ spin valve was cut into 1 × 1 cm^2^ pieces and was patterned into p-STT MTJ with a device size of 1.6 × 1.6 μm^2^ using ion milling and E-beam lithography. Then, p-STT MTJ was passivated, and their contact pads were wire bonded to a sample holder to estimate the electrical characteristics. The magnetic resistance versus applied magnetic field (*R–H*) curve and integration characteristic of the p-STT MTJ were measured with a homemade electrical probing system with a ∼1-T electromagnet using a Keithley 236 source measure unit and an Agilent B2902A semiconductor parameter analyzer.

#### IGZO-Based ReRAM

Five-nanometer-thick indium gallium zinc oxide (IGZO) film was deposited on a 113-nm diameter plug-type TiN-bottom-electrode-patterned wafer by radio frequency (RF) magnetron sputtering at 40 W RF power, 40 sccm Ar flowrate, and 1 sccm O_2_ flowrate for an IGZO target, followed by 400°C annealing for 30 min in N_2_ ambient. For a top electrode patterning, 850 μl photoresist (AZ5214E) was dropped on the IGZO thin film layer followed by spin coating with 5,000 rpm for 30 s and 120°C hard baking for 1 min and 40 s. Then, a photomask with 60 × 60 μm^2^ pattern size was aligned on the substrate followed by exposure to UV light with a beam intensity of 20 mW/cm^2^ for 12 s. The exposed photoresist was developed for 50 s using a developer (AZ300MIF) followed by deionized water rinse for 4 min. Afterward, the top Al electrode was deposited by direct current (DC) magnetron sputtering at 30 W DC power and 30 sccm Ar flowrate for an Al target. Finally, lift-off process was performed to make the top electrode pattern by acetone for 4 min followed by methanol rinse for 4 min and deionized water rinse for 4 min. Thus, the synapse devices have a sandwich device structure of a bottom TiN electrode, an IGZO layer, and a top Al electrode. Electrical characteristic was measured using a Keithley 4200A semiconductor parameter analyzer.

### Pattern Recognition Simulation

#### Neuron

An empirical model was used to simulate the integration characteristic of the p-STT MTJ. The logistic function was used to fit a measured data (Supplementary Figure 1A). Thus, resistance of the p-STT MTJ is given as follows:

(1) $$r\left(n\right)=\frac{r_{\text{min}}-r_{\text{max}}}{1+{\left(\frac{n}{n_{v}+n_{\sigma }}\right)}^{p}}+r_{\text{max}}+r_{\sigma }\;$$

where *n*, *r*_*min*_, *r*_*max*_, *p*, and *n*_*v*_ were the number of applied pulse, minimum and maximum resistance of the p-STT MTJ, fitting constant (=0.3142), and curve fitting parameter depending on the voltage, respectively. The integration characteristic of the p-STT MTJ is determined by *n*_*v*_, which depends on the applied pulse amplitude (Supplementary Figure 1B). In this empirical model, *n*_σ_ and *r*_σ_ were added to account for device variation where *n*_σ_∼*N*(μ_*n*_, σ_*n*_^2^) (μ_*n*_ = 0 and σ_*n*_ = 0.5) and *r*_σ_∼*N*(μ_*r*_, σ_*r*_^2^) (μ_*r*_ = 0 and σ_*r*_ = 0.2) are Gaussian random variables (Supplementary Figures 1C,D).

#### Synapse

In this simulation, IGZO-based ReRAM is used as the artificial synapse, as shown in Supplementary Figure 2A. The IGZO-based ReRAM shows typical bistable current versus voltage (*I*–*V*) curve of interface-type ReRAM, as shown in Supplementary Figure 2B. To emulate synaptic property, we used a synapse model similar to Ziegler et al. (2015) and Hansen et al. (2017). In this model, change in synaptic weight is given by

(2) $$△w_{p,d}\left(t\right)=\beta_{p,d}\left(w\right)w\left(t\right)\left[1-\frac{1}{w_{\text{max}}}w\left(t\right)\right]$$

where *w*, β, and *w*_*max*_ represent the synaptic weight, the weight-dependent learning rate, and maximum synaptic weight, respectively. β determines the potentiation and depression curves depending on the switching mechanism of the ReRAM (Ziegler et al., 2015; Hansen et al., 2017). In order to obtain synaptic weight change, β should be determined. Here, we use a learning rate model given by

(3) $$\begin{array}{c} \beta_{\text{p},\text{d}}\left(△V,△t,w\right)\\ =\{\begin{array}{c} c_{p}\left(△V,△t\right)\times \left(1-\gamma w\right),\;\;\;\mathrm{potentiation}:(V>0)\;\\ -c_{d}\left(△V,△t\right)\times w,\;\;\;\mathrm{depression}:(V<0) \end{array} \end{array}$$

where γ is a positive constant, and *c*_*p*_ and *c*_*d*_ are △*V* and △*t* dependent function. In our model, *c*_*p*_ (=0.275) and *c*_*d*_ (=0.063) are constant since △*V* and △*t* were fixed for the potentiation and depression. The simulation is well correlated with potentiation/depression of the experimental data, as shown in Supplementary Figure 2C.

#### Synaptic Weight Update

We used simplified spike timing-dependent plasticity (STDP) learning rule for training SNN. Synaptic weight was updated with the following equation:

(4) $$\text{w}\left(t_{n+1}\right)=\left\{ \begin{array}{c} w\left(t_{n}\right)+△w_{\text{p}}\left(t_{n}\right),0\leq t_{\text{post}}-t_{\text{pre}}<10T\\ w\left(t_{n}\right)+△w_{\text{d}}\left(t_{n}\right),otherwise \end{array}\right.$$

where △ *w*_p_ and △ *w*_nd_ are the synaptic weight change for the potentiation and depression, respectively. *T* is the time of a one cycle of integration–read–reset. Since we assumed a synchronous system, *T* is constant. Additional circuits are required for STDP operation. However, it is beyond the scope of this paper to deal with synaptic learning circuit in detail. When the spiking time difference between a preneuron (*t*_pre_) and a postneuron (*t*_post_) was <10 cycles (1 cycle = integration–read–reset), the synapses connected with the pre- and postneurons were potentiated, and the remaining synapses were depressed.

## Results

### Artificial Neural Network Based on p-STT-Based Neuron

**FIGURE 1:**
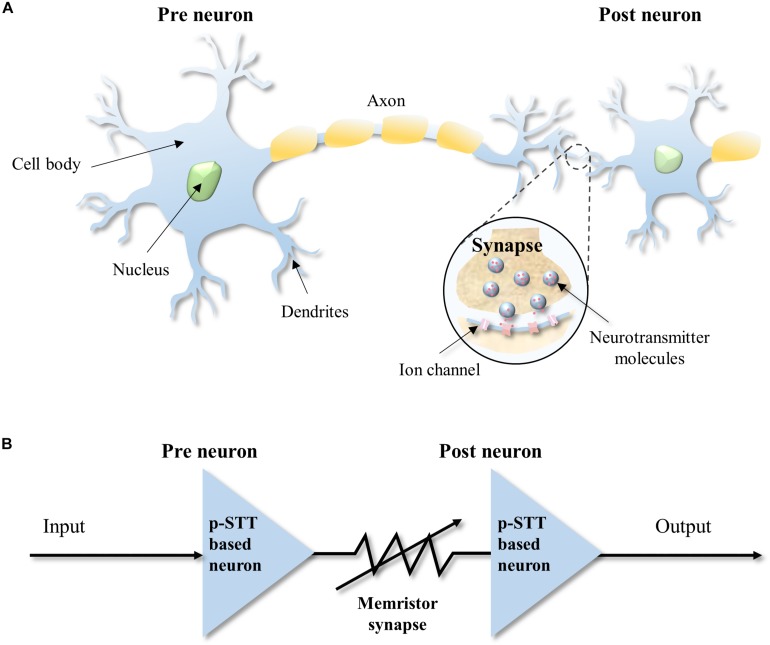
Schematic of neural network. **(A)** Biological neural network. **(B)** Artificial neural network using the perpendicular spin transfer torque (p-STT)-based neurons and memristor synapse.

In biological neural networks, neurons are connected to other neighboring neurons via synapses, as shown in Figure 1A. Neurons integrate input spike signals from adjacent neurons via synapses, i.e., integrate. In addition, neurons generate output spike signals when membrane potentials reach a threshold value, i.e., fire. This neuronal behavior is called “integrate-and-fire,” which is the key operation of neuron (Hodgkin and Huxley, 1952; Izhikevich, 2003)**_._** Similarly, artificial neurons could be connected with other artificial neurons via artificial synapses, where p-STT-based neurons are connected with memristor-type synapse, as shown in Figure 1B. The p-STT-based neurons receive spike signals through synapses connected with preneurons, integrate the signals, and then sends out output spike signals when the resistance of the p-STT-based neurons reaches a certain threshold value. In the following sections, we will describe in detail how p-STT-based neuron works.

### Magnetic Properties of p-MTJ

**FIGURE 2:**
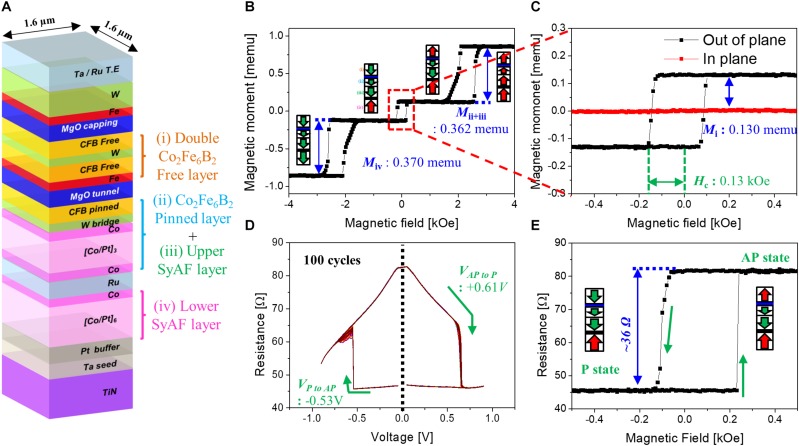
Magnetic and electrical properties of the perpendicular spin transfer torque (p-STT)-based neuron (1.6× 1.6 μm^2^). **(A)** Schematic structure. **(B)** *M*–*H* curve in a wide scanning range of the applied perpendicular magnetic field (i.e., −4 ∼ + 4 KOe). **(C)** *M*–*H* curve in a narrow scanning range of the applied perpendicular magnetic field (i.e., −0.5 ∼ + 0.5 KOe). **(D)** *R*–*V* curve. **(E)** *R*–*H* curve of the p-STT-based neuron.

Figure 2A shows schematic structure of p-STT MTJ. Its magnetic moment versus applied perpendicular magnetic field (*M–H*) loop was investigated to determine the static magnetic behavior of the p-STT MTJ, as shown in Figures 2B,C. It includes four perpendicular magnetic anisotropy (PMA) layers: a double Co_2_Fe_6_B_2_ free layer (i in Figure 2A), Co_2_Fe_6_B_2_ pinned layer (ii in Figure 2A), upper [Co/Pt]_3_ SyAF layer (iii in Figure 2A), and lower [Co/Pt]_6_ SyAF layer (iv in Figure 2A). Here, the Co_2_Fe_6_B_2_ pinned layer was ferrocoupled with the upper SyAF layer, whereas the upper [Co/Pt]_3_ SyAF layer was antiferro coupled with the lower [Co/Pt]_6_ SyAF layer. The magnetic moments of the double Co_2_Fe_6_B_2_ free layer, Co_2_Fe_6_B_2_ pinned layer ferrocoupled with the upper [Co/Pt]_3_ SyAF layer, and lower [Co/Pt]_6_ SyAF layer were 0.130 (*M*_*i*_ in the inset of Figure 2C), 0.362 (*M*_*ii* + *iii*_ in Figure 2B), and 0.370 (*M*_*iv*_ in Figure 2B) memu, respectively. In addition, the double Co_2_Fe_6_B_2_ free layer showed an excellent interface PMA characteristic with a good squareness and fair coercivity (*H*_*c*_, ∼0.13 kOe), as shown in Figure 2C. This result indicates that the MgO tunneling barrier had good face-centered cubic crystallinity that enhanced the coherent tunneling of the spin electrons (Lee et al., 2016a,c,d). The magnetic resistance versus voltage (*R–V*) behavior at room temperature (295 K) was measured to investigate the spin transfer torque switching behavior of the p-MTJ, as shown in Figure 2D. The switching voltage from parallel to antiparallel states was −0.53 *V* (*V*_*PtoAP*_), while the switching voltage from antiparallel to parallel states was + 0.61 *V* (*V*_*APtoP*_). The magnetic resistance versus magnetic field (*R–H*) loop of the p-STT MTJ is shown in Figure 2E. When the applied perpendicular magnetic field was scanned from + 0.5 to −0.5 kOe, the electron spin direction of the double Co_2_Fe_6_B_2_ free layer was rotated from upward to downward so that the electron spin directions between the double Co_2_Fe_6_B_2_ free and pinned layers were switched from antiparallel to parallel states. As a result, the resistance of the p-STT MTJ decreased from 82 to 46 Ω. The squareness and coercivity of the p-STT MTJ measured with an *R–H* loop was almost the same as that measured with an *M–H* loop, indicating that this device could maintain a stable magnetic state in a zero magnetic field so that the integration behavior would be characterized during the switch from parallel to antiparallel between the double Co_2_Fe_6_B_2_ free and pinned layers.

### Integration Property of p-MTJ Spin Valves

**FIGURE 3:**
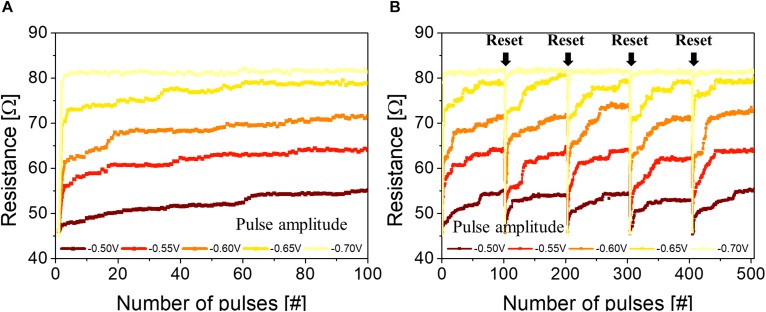
Integration characteristics of the perpendicular spin transfer torque (p-STT) magnetic tunneling junction (MTJ)-based neuron. **(A)** Dependence of the integration behavior on the input spike number and amplitude. **(B)** Repeated integration characteristic of the p-STT MTJ (five sets of 100 input pulse spikes).

**FIGURE 4:**
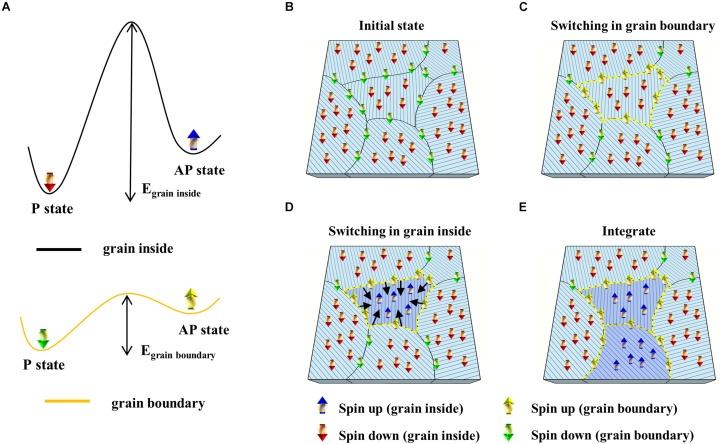
Integration mechanism of the perpendicular spin transfer torque (p-STT) magnetic tunneling junction (MTJ). **(A)** Schematic of switching energy diagram at grain inside (black) and grain boundary (yellow). Schematic illustration of integration mechanism: **(B)** initial state, **(C)** switching at grain boundary, **(D)** switching at grain inside, and **(E)** integration.

Interestingly, the p-STT MTJ showed integration property when consecutive voltage pulses (spike) were applied, as shown in Figure 3A. The spike width was 50 μs, and the spike amplitude was varied from −0.50 to −0.70 V. At all spike amplitudes, i.e., −0.50, −0.55, −0.60, −0.65, and −0.70 V, the p-STT MTJ performed the integration at input spikes of ∼100 pulses. In addition, the resistance difference increased when the input spike amplitude increased from −0.50 to −0.70 V at input spikes of ∼100 pulses, as shown in Figure 3A. Over an input spike amplitude of −0.7 V, no integration behavior was found. In addition, the p-STT MTJ showed a good repeatability for five sets of ∼100 input spike pulses, where the resistance increment by the 100 input spike pulses increased with the input spike, as shown in Figure 3B. Our proposed p-STT MTJ in Figure 3 showed a unique neuron characteristic (i.e., integration characteristic) compared to MTJ-based neurons (stochastic characteristic with a two-terminal device or leaky-integrate-and-fire characteristic with a three-terminal device), as shown in Supplementary Table 1. The mechanism of this behavior could be explained by understanding the grain-size distribution of the polycrystalline MgO tunneling barrier. The distribution of the sputtered polycrystalline MgO tunneling barrier was 0.6 to ∼1.8 nm, where the average grain size was ∼0.94 nm, as shown in Supplementary Figure 3. This indicates that even for a p-STT MTJ with a cell size of 35×35 nm^2^, multiple grains would exist within the p-STT MTJ cell, as shown in Supplementary Figure 4. As a result, we can expect that the p-STT MTJ with a cell size of 35× 35 nm^2^ would show an integrate characteristic similar to Figure 3 since it has a large number of grain within the p-STT MTJ cell. The interfacial PMA of both the double Co_2_Fe_6_B_2_ free and pinned layers originated from the hybridization between O atoms and X (Fe or Co) atoms at the MgO tunneling barrier and Co_2_Fe_6_B_2_ layer interface. Thus, the polygrain size distribution of the polycrystalline MgO tunneling barrier directly and strongly affects the ferromagnetic properties of both the double Co_2_Fe_6_B_2_ free and pinned layers, i.e., resistance difference between parallel and antiparallel states of the p-MTJ. In addition, the hybridized Fe–O and Co–O bonds within the grains would be well oriented with the crystallinity of the MgO tunneling barrier, so the electron spins would require a high activation energy to switch from parallel to antiparallel. Otherwise, the spin electrons at the grain boundaries would have a relatively low energy barrier to switch from parallel to antiparallel, compared with the spin electrons within the grains (MacLaren and Willoughby, 2001; Victora et al., 2003; Kondo et al., 2018), as shown in Figure 4A. Thus, the spin electrons at the grain boundaries (Figure 4B) would first be switched from parallel to antiparallel states (Figure 4C), and the spin electrons inside the grain would then rotate due to the ferrocoupling between the spin electrons at the grain boundary and inside the grain (Figure 4D). As a result, the spin electrons in the grains would be switched from parallel to antiparallel, which would be a similar switching behavior to a previous report (Suzuki et al., 2016). This switching process would induce the integration behavior when the spikes are sequentially applied to p-STT MTJ (Figure 4E). The integration behavior of a p-STT MTJ was influenced by the crystallinity of the MgO tunneling barrier in Figure 2A, i.e., a better crystallinity of the MgO tunneling barrier led to a better integration characteristic, as shown in Supplementary Figure 5. This integration behavior of the p-STT MTJ would suggest that the p-STT MTJ could be applied with the complementary metal–oxide–semiconductor field-effect transistor (C-MOSFET) technology to produce artificial neuron. In general, the perpendicular spin torque switching time of a p-STT MTJ has been reported as ∼10 ns, which is the fastest switching time among other semiconductor devices (Hu G. et al., 2018). In addition, the operation of the integration by a p-STT MTJ in Figure 3 was performed prior to a full the perpendicular spin torque switching. Thus, the width of a spike pulse in Figure 3 could be less than ∼10 ns if the size of a neuron using a p-STT MTJ can be scaled down–up to 35 × 35 nm^2^, suggesting a lowest power consumption per a spike in neuron (i.e., 1.6 × 1.6 μm^2^), as shown in Supplementary Table 2.

### p-STT MTJ-Based Integrate-and-Fire Neuron

**FIGURE 5:**
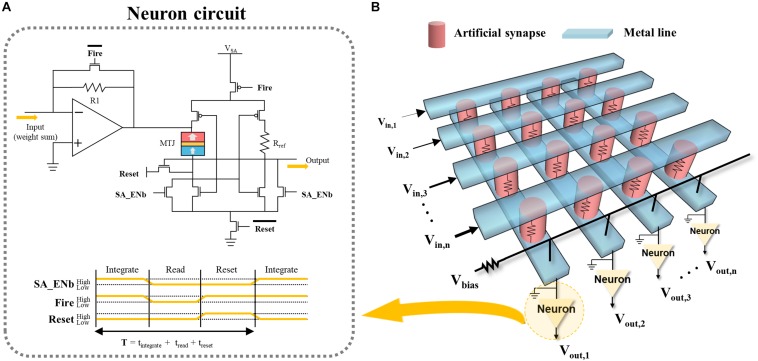
Schematic of artificial neural network. **(A)** Crossbar array of artificial synapses and **(B)** neuron circuit for integrate-and-fire.

Although the p-STT MTJ exhibited integration behavior depending on the input spike amplitude, it requires an additional circuit to perform the fire operation. Thus, the p-STT MTJ-based neuron circuit was designed using one p-STT MTJ, seven n-MOS FETs, three p-MOS-FETs, and one reference resistance to conduct the integrate-and-fire operation as shown in Figure 5A. Note that we calculated the area of the p-STT MTJ-based integrate-and-fire neuron using 1.6×1.6μm^2^ p-STT MTJ (i.e., ∼8.2 μm^2^), which was approximately one-fourth smaller than the previous report (Sourikopoulos et al., 2017), as shown in Supplementary Figure 6 and Supplementary Table 3. In this circuit, “fire” occurs when the resistance of the p-STT-based neuron exceeds the reference resistance (*R*_*ref*_). The neuron receives control signals from a controller and performs integration, read, and reset operations in each clock cycle, as shown in Figure 5A. One controller can control multiple neurons simultaneously. In order to implement neural network, cross-point array can be used to realize analog matrix-vector multiplication. Figure 5B shows the schematic illustration of typical cross-point neural network implementation, which was fabricated by a cross-point synapse array being connected with our proposed p-STT MTJ neuron. Synapse would be IGZO-based memristor (in our experiment shown in Supplementary Figure 2). Where the bias voltage (*V*_*bias*_) serves to ensure that the p-STT-based neuron is within its proper operating range.

### Pattern Recognition

**FIGURE 6:**
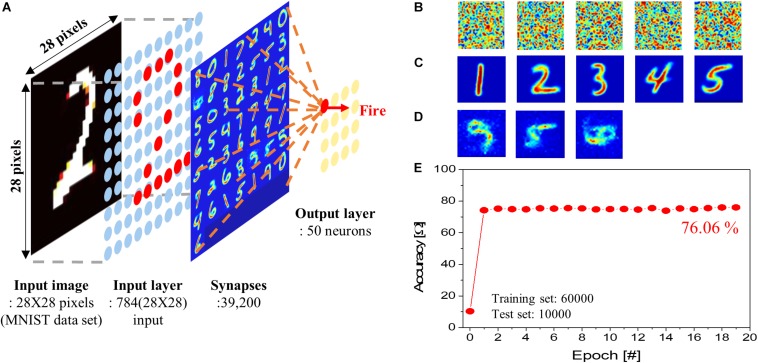
Pattern recognition simulation. **(A)** Schematic of a single-layer spiking neural network (SNN). **(B)** Normalized synaptic weight before learning. **(C)** Normalized synaptic weight connected with active neurons after learning. **(D)** Normalized synaptic weight connected with silent neurons after learning. **(E)** Pattern recognition accuracy.

To investigate the performance of the SNN, a single-layer SNN consisting of input and output layers (50 p-STT-based neuron) was designed, as shown in Figure 6A. In this simulation, IGZO-based ReRAM was used in artificial synapse. A performance test of the SNN was carried out using the MNIST handwritten image set. MNIST images (6 × 10^4^) were used for training, and 1 × 10^4^ images not included in the training were used for testing. The probability of the input spike occurrence was set to be proportional to the pixel value of an input image, and the amplitude of an input spike was set to −1 V. The neurons integrate the input spike signals and fire when the resistance of the p-STT MTJ exceed *R*_*th*_ (=70 Ω). When the neurons fire, they generated an output spike. The winner takes all (WTA) was applied to the output neuron nodes. WTA improved the accuracy of a single-layer SNN since the WTA guarantees non-linear mapping in a single-layer SNN (Du et al., 2015; Hansen et al., 2017). Finally, only the synaptic weights associated with the fired output neurons were updated. In the initial synaptic weight map, the conductance of the synapses was randomly distributed. After training, the distribution of synaptic weights was changed. The weights for active and silent neurons are shown in Figures 6C,D, respectively. Even if there were more than 10 epochs, there were some silent neurons, as shown in Figure 6D. These silent neurons exhibited almost no firing during training. The reason for this is that the WTA updates only synaptic weights associated with neurons that have fired; consequently, synaptic weights connected with neurons that rarely fire are slower to learn. As a result, these less learned synapses reduce the firing rate of the connected silent neurons compared to other neurons. In the end, learning is rarely achieved for the silent neurons. In biological neural networks, there is a mechanism called “homeostasis” to overcome these problems. With this mechanism, a neuron that frequently fires increases the threshold required to fire, and a neuron that rarely fires decreases it (Lee et al., 2016b,d; Johnson et al., 2018). This mechanism lowers the fire threshold of neurons where learning has not been achieved; thus, it causes neurons to be more likely to fire during subsequent learning. However, it is difficult to change the reference resistance *R*_*th*_ once it is set in the circuit. This remains a problem to be solved in the future. We use simplified STDP learning rule for synaptic learning. The synaptic weights before training are shown in Figure 6B. First, we simulated the dependence of pattern recognition accuracy on read error using our proposed the cross-point synapse array (i.e., Figure 5B) being connected with our proposed p-STT MTJ neuron (i.e., Figure 5A), as shown in Supplementary Figure 7. The pattern recognition accuracy sustained at ∼76% up to read error of 5% and then rapidly decreased with read error larger than 5%. In addition, we tested the dependence of pattern recognition accuracy on the reference resistance by simulation, as shown below Supplementary Figure 8. We determined the reference resistance that showed the highest accuracy of pattern recognition simulation. Using the simulated reference resistance, the pattern recognition accuracy rapidly increased to ∼76% in two epochs, as shown in Figure 6E. Since the single-layer SNN used in training is learned through STDP unsupervised learning, so only clustering was performed for each output stage. Therefore, the most frequent output values of each node were compared with the determined input value to measure the pattern recognition accuracy. The single-layer SNN, composed of p-STT-based neurons, showed a maximum recognition accuracy of ∼76%, which was somewhat lower than that of other reported neural networks (Burr et al., 2014). In the single-layer SNN, pattern recognition accuracy increases with the number of output neurons (Querlioz et al., 2015; Zahari et al., 2015; Hansen et al., 2017). However, even if the number of output neurons is increased to 100, it is difficult to obtain more than 90% accuracy. The major reason for the low accuracy is the lack of proper learning algorithms to train SNN. The spike signals are not differentiable, so global learning rule such as backpropagation cannot be used for training SNN. Therefore, local learning rule such as STDP is mainly used for training SNN. This limits the structure of neural network to a single layer. Therefore, in order to increase the accuracy of the SNN, further study of the learning algorithm is necessary.

## Discussion

p-STT MTJ could perform integration when the spin electron directions at double Co_2_Fe_6_B_2_ free and pinned layers were switched from parallel to antiparallel states. However, for the integrate-and-fire operation, a neuron circuit performing the fire behavior was essentially designed. Pattern recognition accuracy of ∼76% was achieved using a ReRAM-based synapse model and the STDP learning rule. In summary, the p-STT-based neuron could perform like a typical neuron showing integrate-and-fire behavior and would be a suitable for SNN. In addition, a cross-point synapse array is essentially necessary, where a selector is vertically stacked on a synapse to eliminate a sneak current between synapses. Thus, further studies are necessary on processes for fabricating cross-point synapse arrays connected with p-STT-based neurons. In addition, since the two-terminal p-STT-based neuron can perform only the integration behavior, a circuit performing the fire behavior should also be designed. Therefore, further study is also necessary on a three-terminal p-STT-based neuron that uses a magnetic domain moving mechanism. Finally, since a strong merit of the p-STT-based neuron would be its power consumption; further study is necessary for a neuron circuit design with low power consumption.

## Data Availability Statement

The datasets generated for this study are available on request to the corresponding author.

## Author Contributions

JP conceived and designed the study. JC, HJ, KA, and JB fabricated the samples and carried out measurements. JK and WY designed the neuron circuit. DK performed the simulations with the help of JP. All authors contributed to discussions regarding the research. DK, JC, and JP wrote the manuscript.

## Conflict of Interest

The authors declare that the research was conducted in the absence of any commercial or financial relationships that could be construed as a potential conflict of interest.
