# Supplementary material for: Double MgO-Based Perpendicular Magnetic Tunnel Junction for Artificial Neuron
Source: Front Neurosci. 2020 Apr 30;14:309. doi: 10.3389/fnins.2020.00309 (PMC7204637; doi:10.3389/fnins.2020.00309)
Supplement: Supplementary file 1 [file Presentation_1.pptx]

## Slide 1
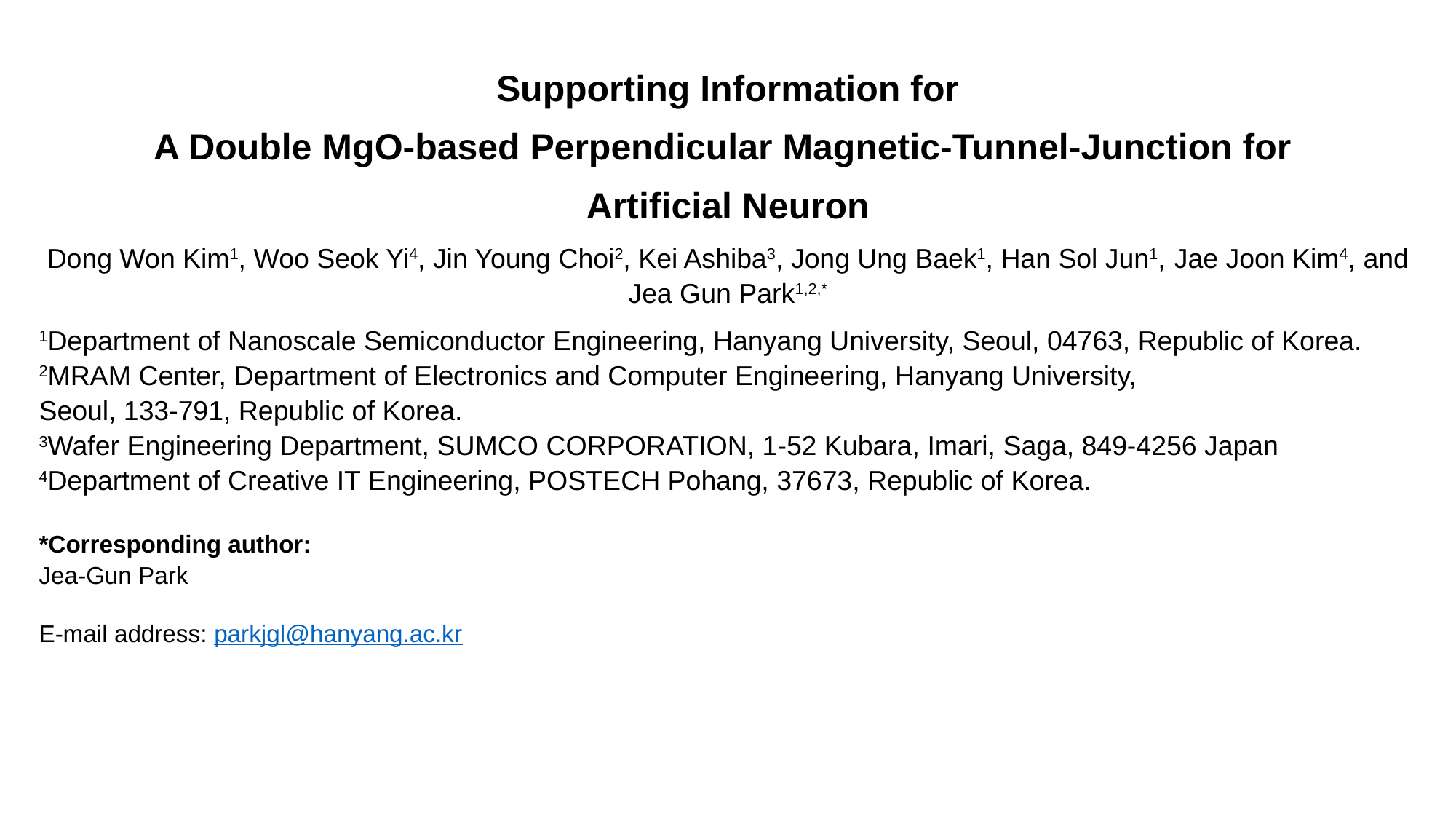

Supporting Information for
A Double MgO-based Perpendicular Magnetic-Tunnel-Junction for
Artificial Neuron
Dong Won Kim1, Woo Seok Yi4, Jin Young Choi2, Kei Ashiba3, Jong Ung Baek1, Han Sol Jun1, Jae Joon Kim4, and Jea Gun Park1,2,*
1Department of Nanoscale Semiconductor Engineering, Hanyang University, Seoul, 04763, Republic of Korea.
2MRAM Center, Department of Electronics and Computer Engineering, Hanyang University,
Seoul, 133-791, Republic of Korea.
3Wafer Engineering Department, SUMCO CORPORATION, 1-52 Kubara, Imari, Saga, 849-4256 Japan
4Department of Creative IT Engineering, POSTECH Pohang, 37673, Republic of Korea.
*Corresponding author:
Jea-Gun Park
E-mail address: parkjgl@hanyang.ac.kr

## Slide 2
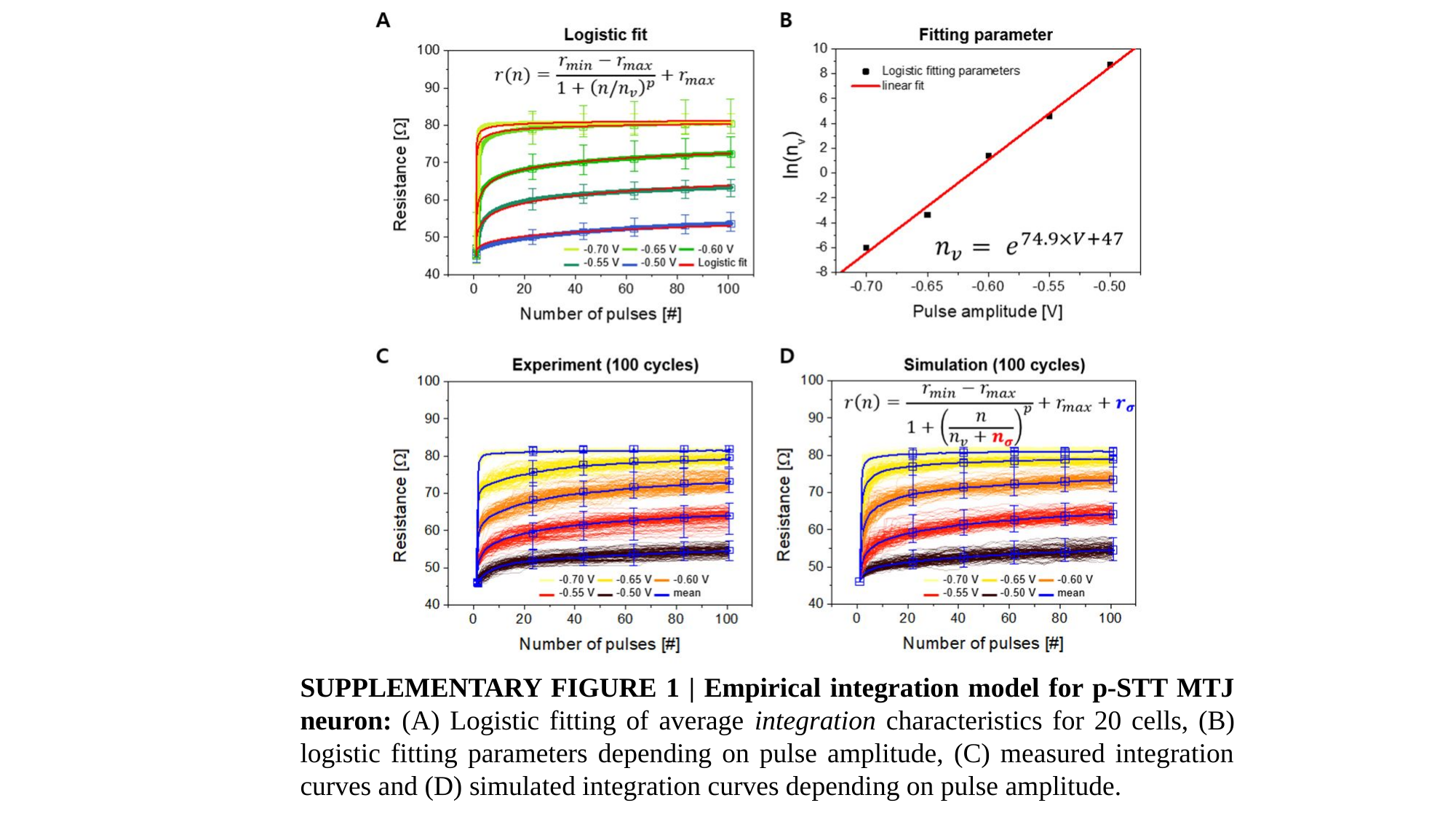

SUPPLEMENTARY FIGURE 1 | Empirical integration model for p-STT MTJ neuron: (A) Logistic fitting of average integration characteristics for 20 cells, (B) logistic fitting parameters depending on pulse amplitude, (C) measured integration curves and (D) simulated integration curves depending on pulse amplitude.

## Slide 3
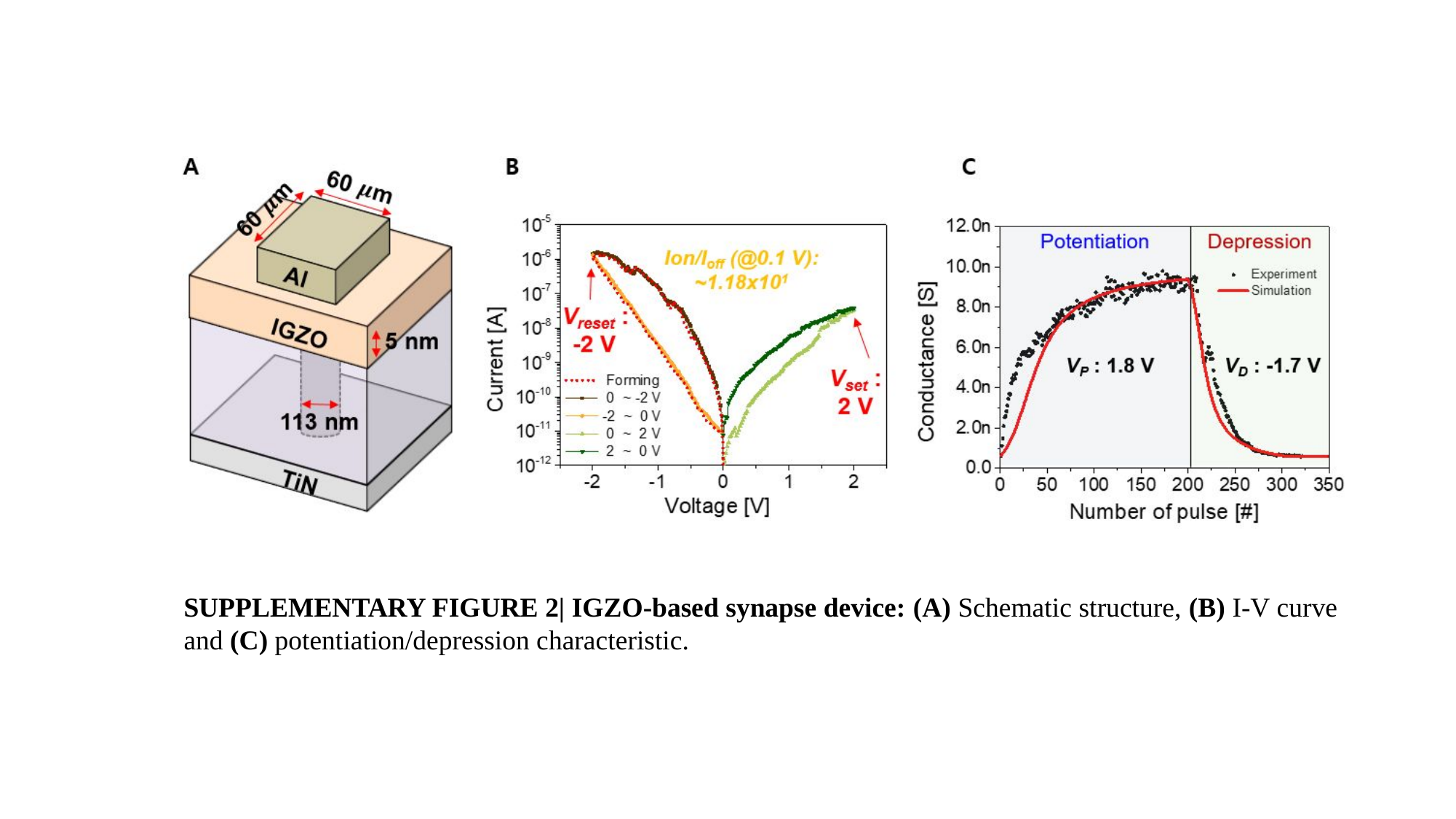

SUPPLEMENTARY FIGURE 2| IGZO-based synapse device: (A) Schematic structure, (B) I-V curve and (C) potentiation/depression characteristic.

## Slide 4
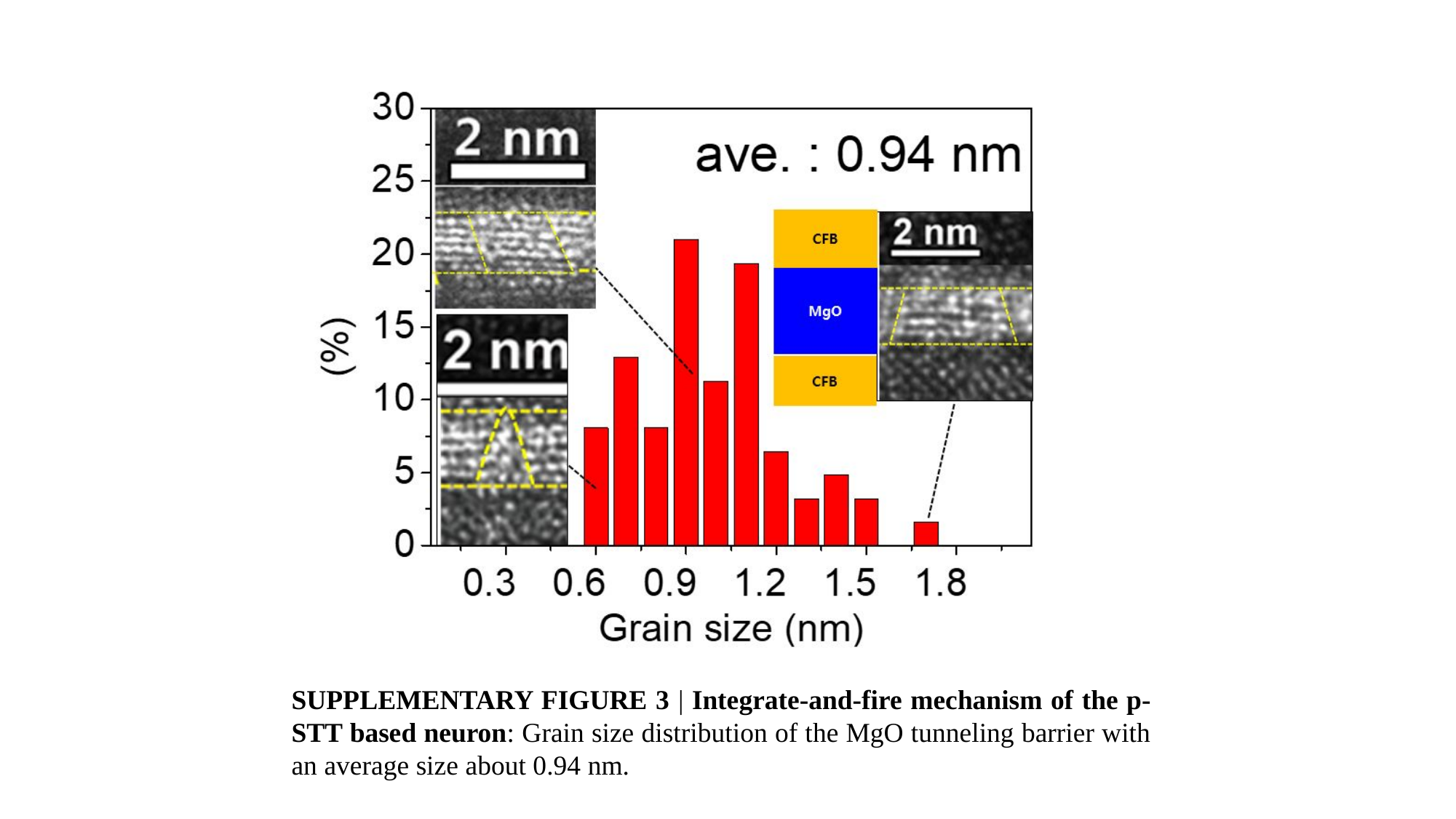

SUPPLEMENTARY FIGURE 3 | Integrate-and-fire mechanism of the p-STT based neuron: Grain size distribution of the MgO tunneling barrier with an average size about 0.94 nm.

## Slide 5
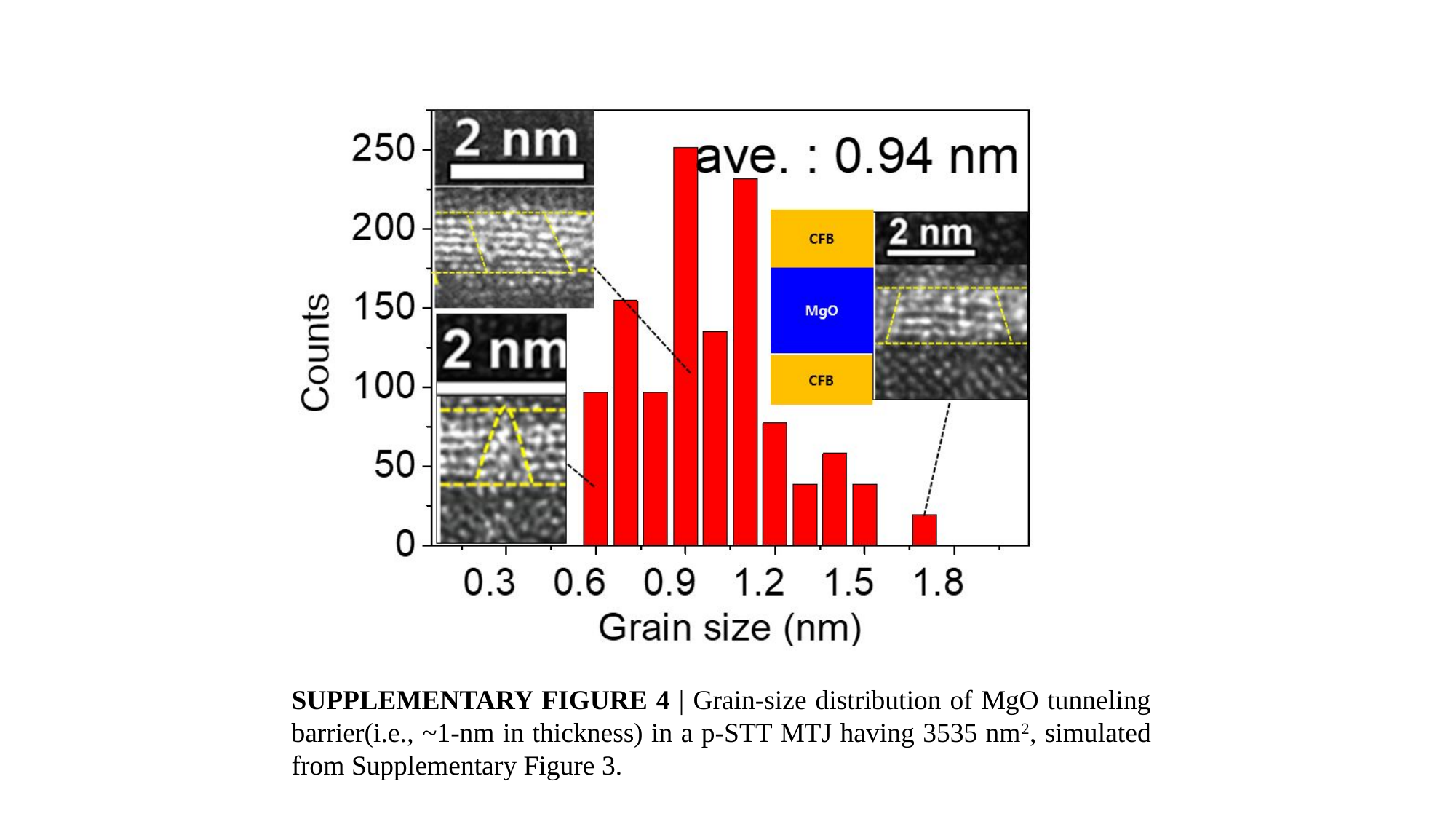

## Slide 6
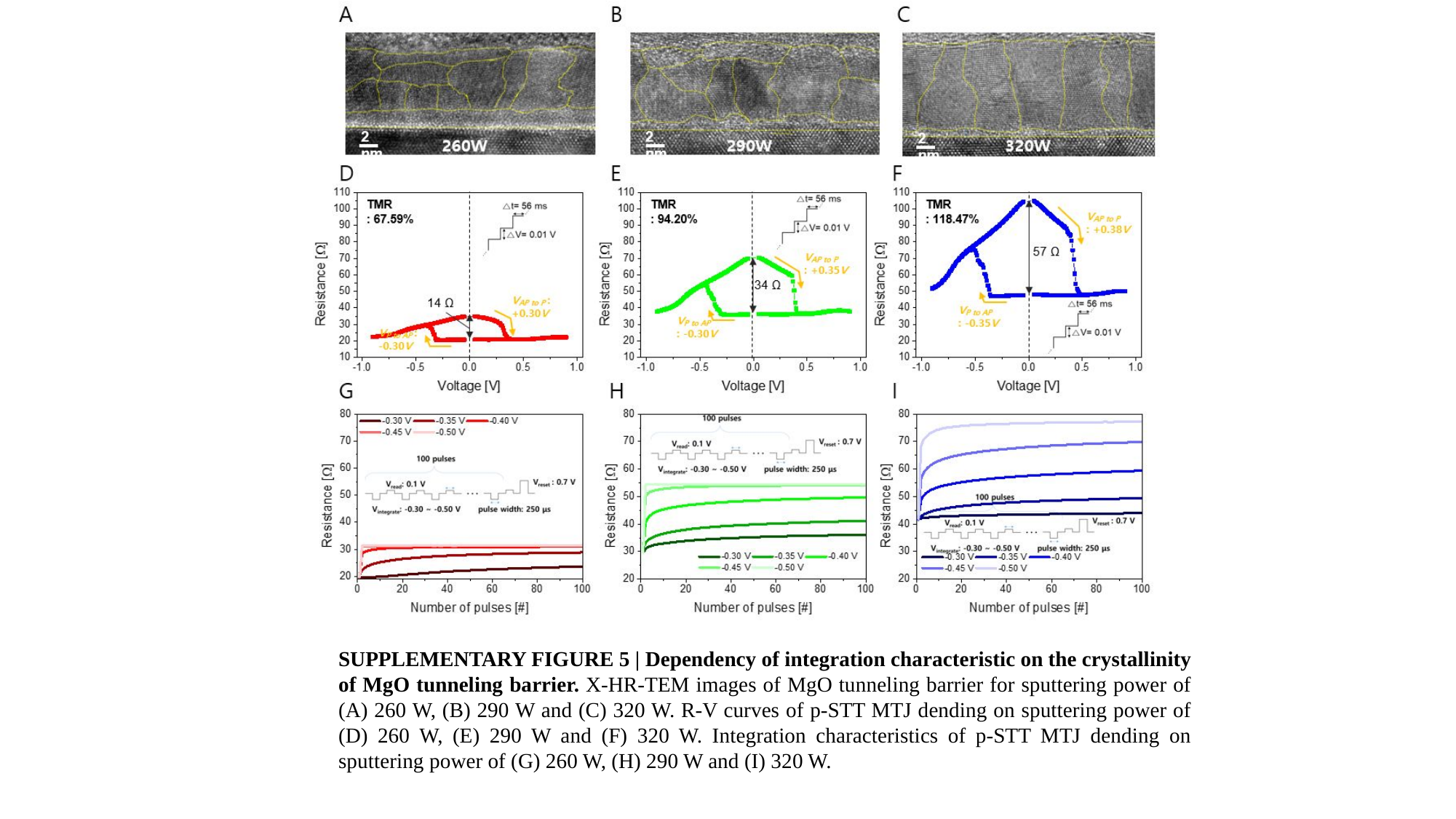

SUPPLEMENTARY FIGURE 5 | Dependency of integration characteristic on the crystallinity of MgO tunneling barrier. X-HR-TEM images of MgO tunneling barrier for sputtering power of (A) 260 W, (B) 290 W and (C) 320 W. R-V curves of p-STT MTJ dending on sputtering power of (D) 260 W, (E) 290 W and (F) 320 W. Integration characteristics of p-STT MTJ dending on sputtering power of (G) 260 W, (H) 290 W and (I) 320 W.

## Slide 7
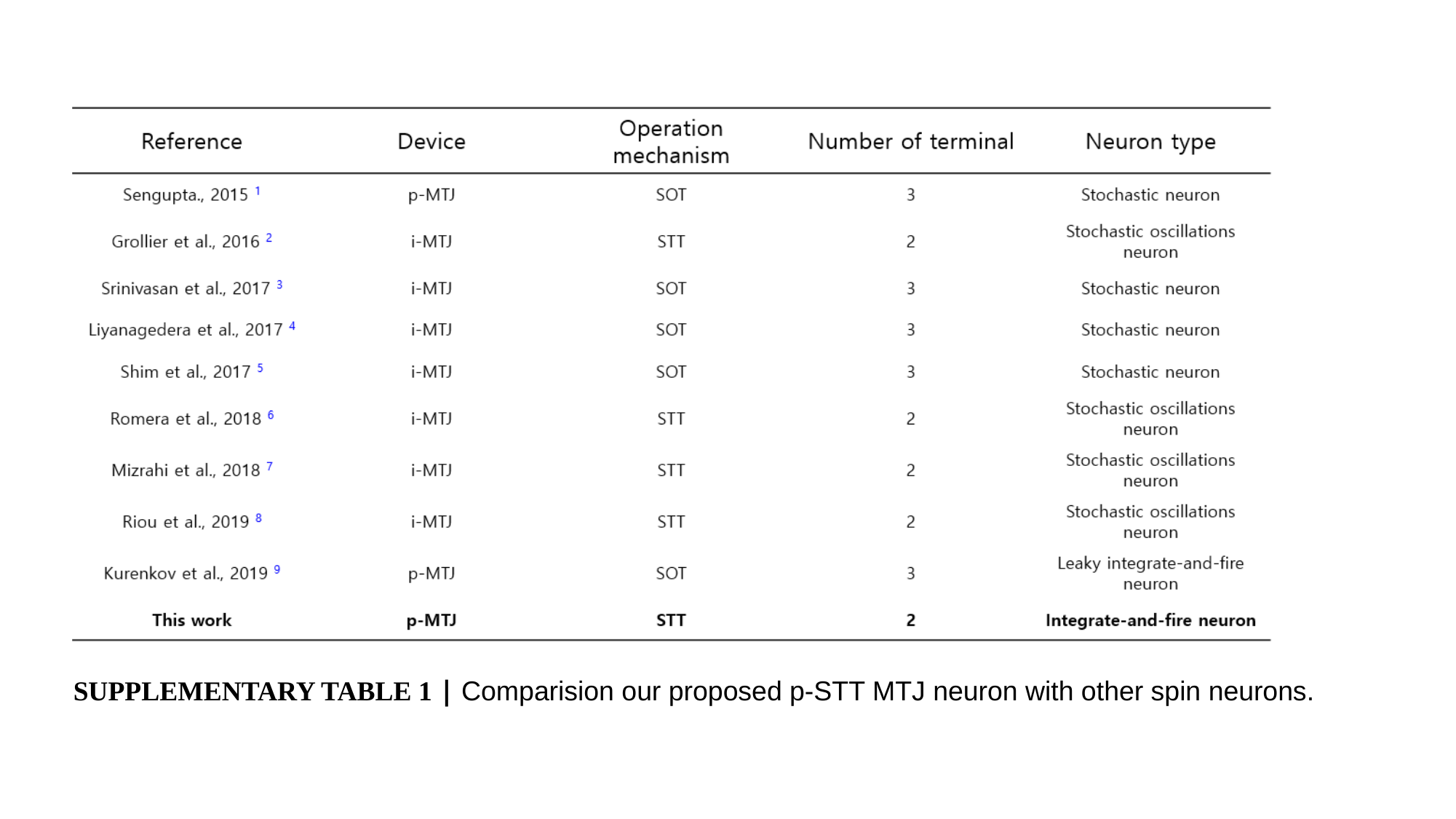

SUPPLEMENTARY TABLE 1 | Comparision our proposed p-STT MTJ neuron with other spin neurons.

## Slide 8
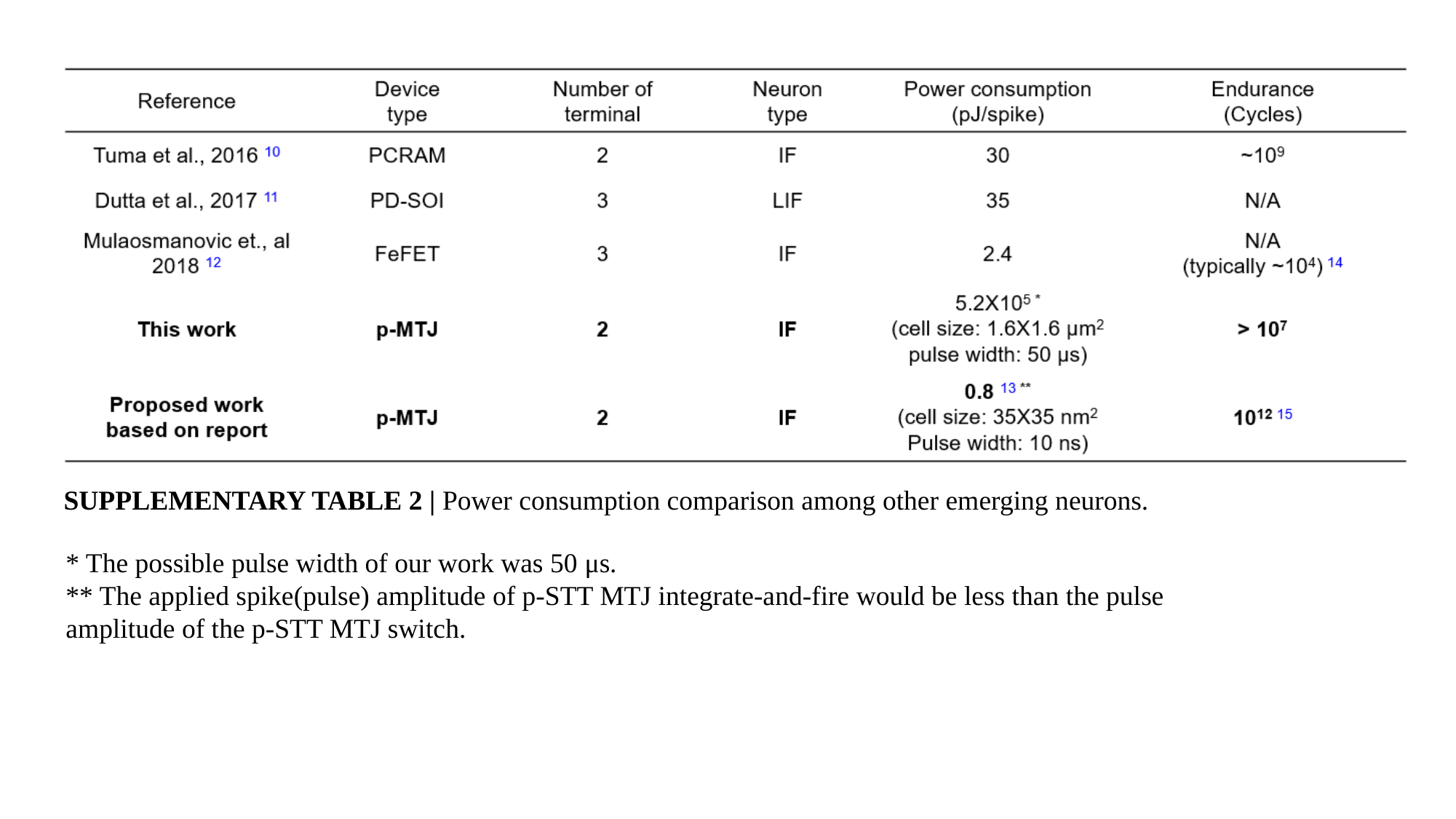

SUPPLEMENTARY TABLE 2 | Power consumption comparison among other emerging neurons.
* The possible pulse width of our work was 50 μs.
** The applied spike(pulse) amplitude of p-STT MTJ integrate-and-fire would be less than the pulse amplitude of the p-STT MTJ switch.

## Slide 9
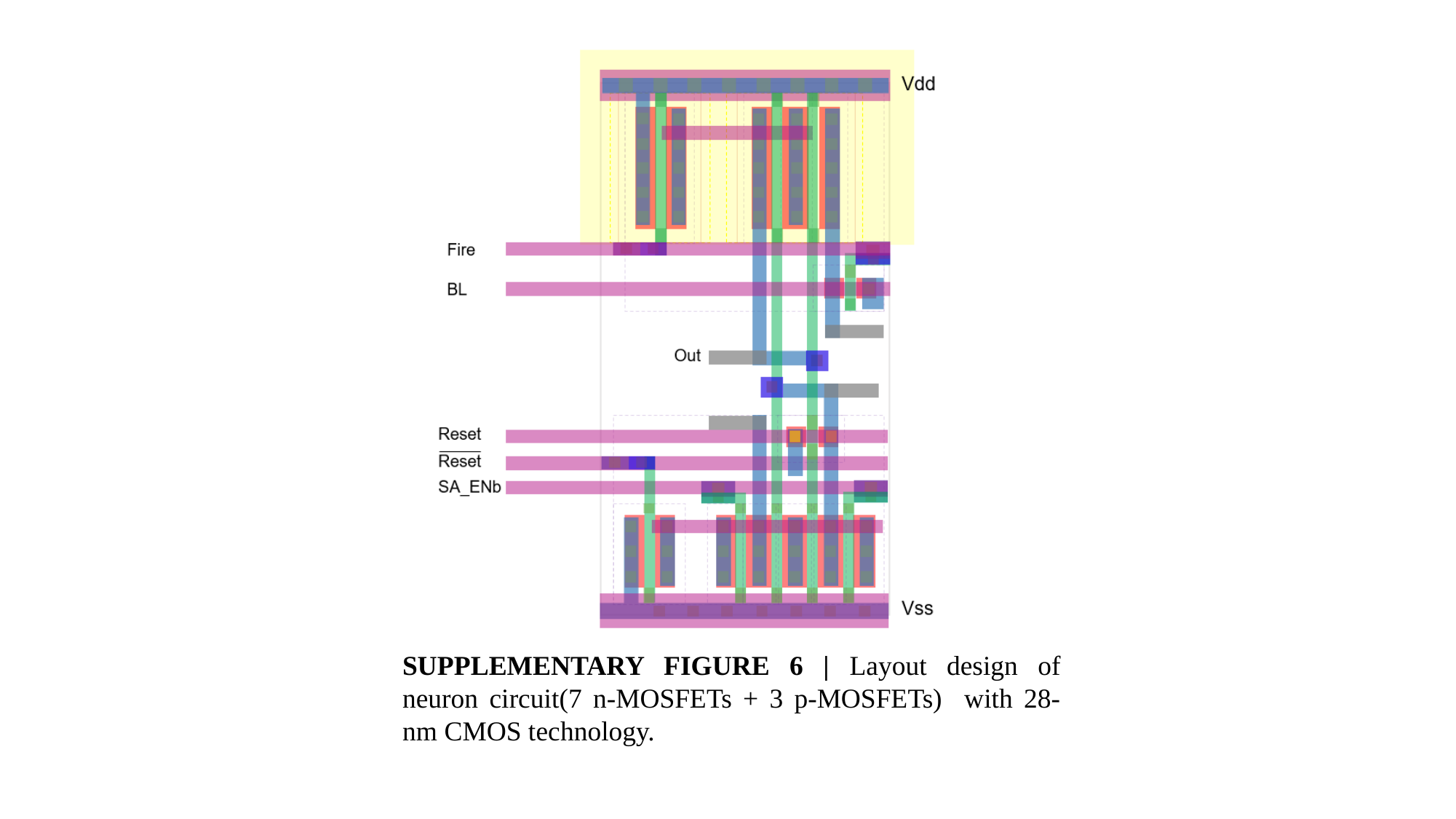

SUPPLEMENTARY FIGURE 6 | Layout design of neuron circuit(7 n-MOSFETs + 3 p-MOSFETs) with 28-nm CMOS technology.

## Slide 10
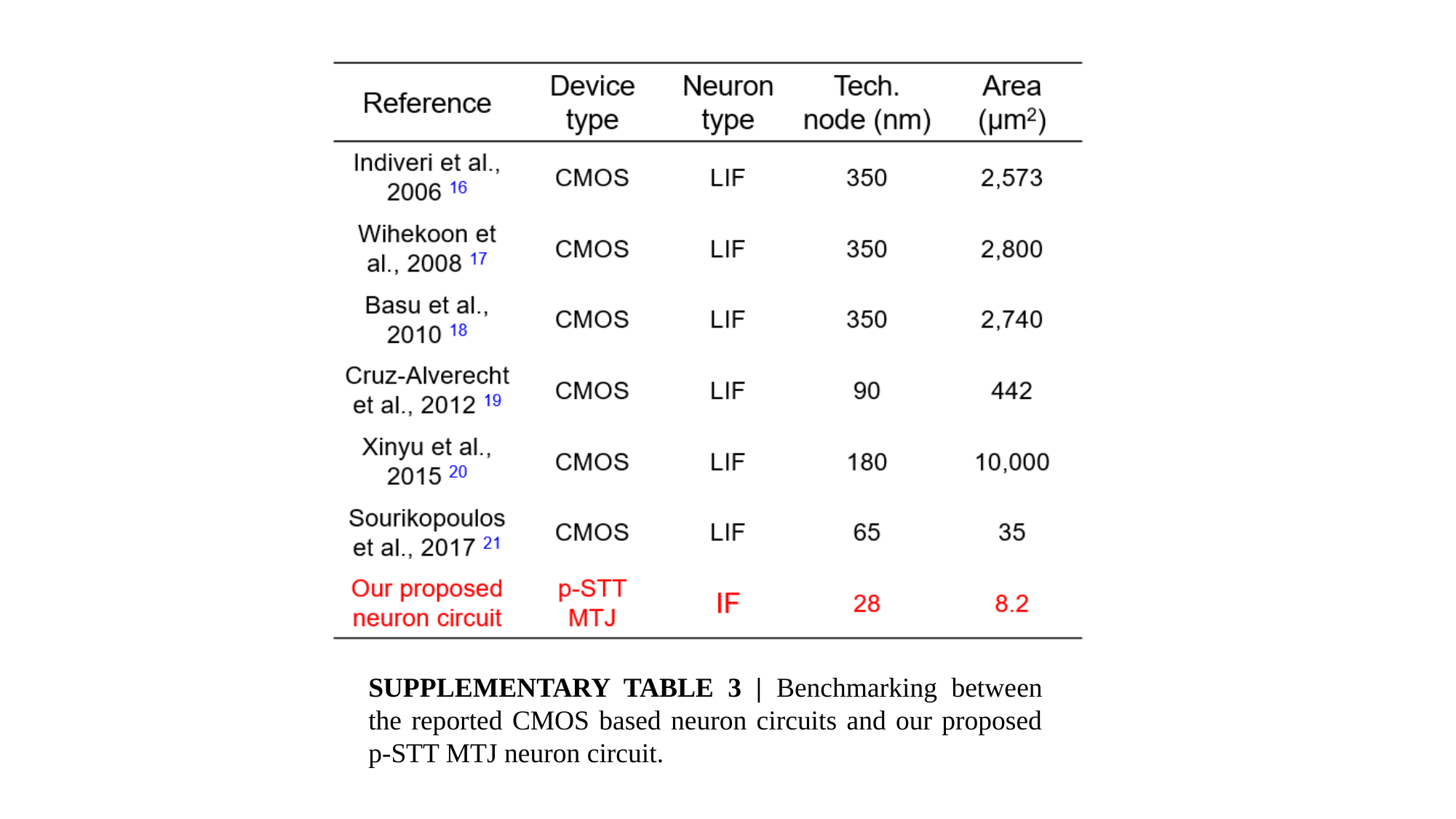

SUPPLEMENTARY TABLE 3 | Benchmarking between the reported CMOS based neuron circuits and our proposed p-STT MTJ neuron circuit.

## Slide 11
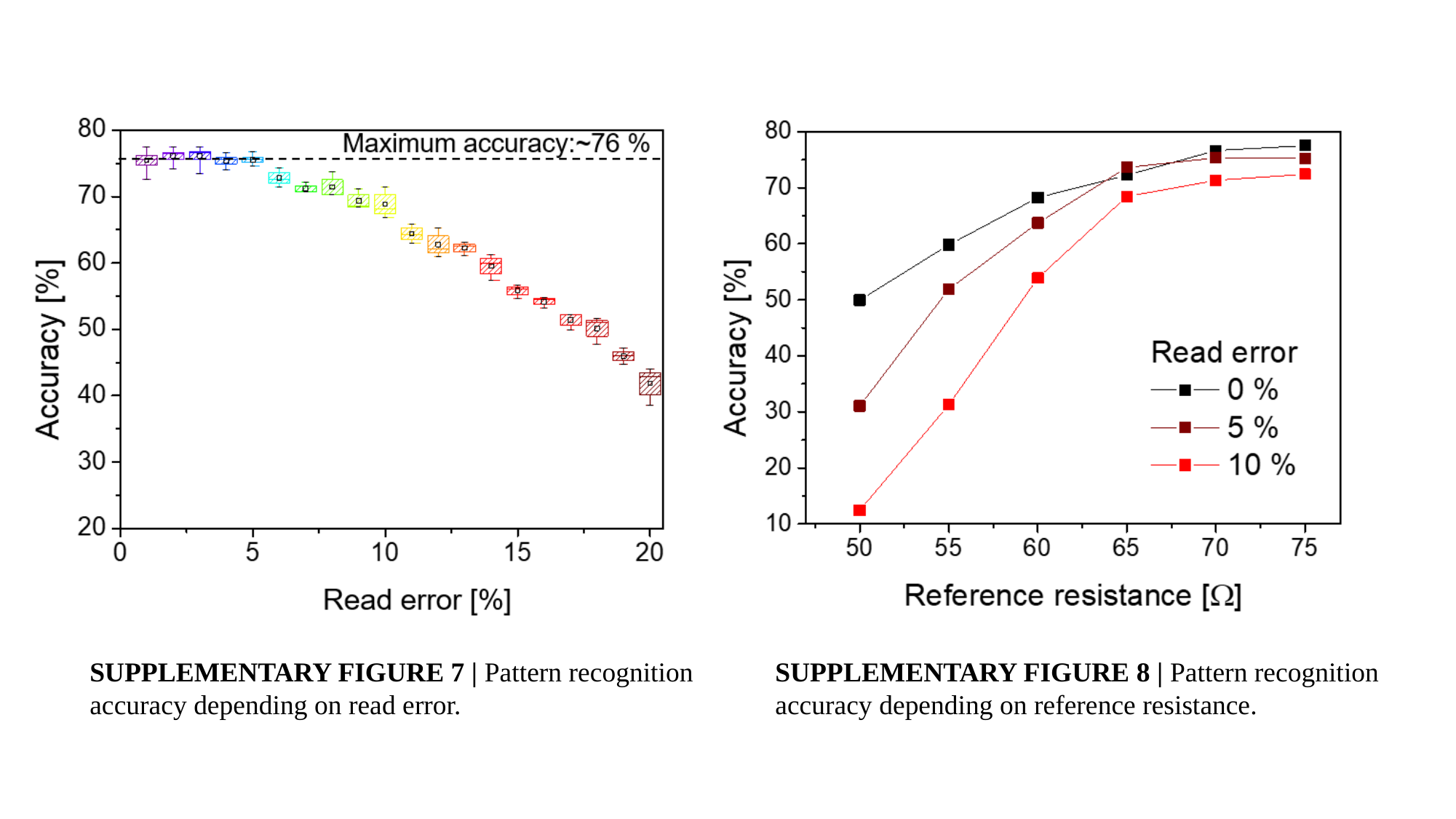

SUPPLEMENTARY FIGURE 7 | Pattern recognition accuracy depending on read error.
SUPPLEMENTARY FIGURE 8 | Pattern recognition accuracy depending on reference resistance.

## Slide 12
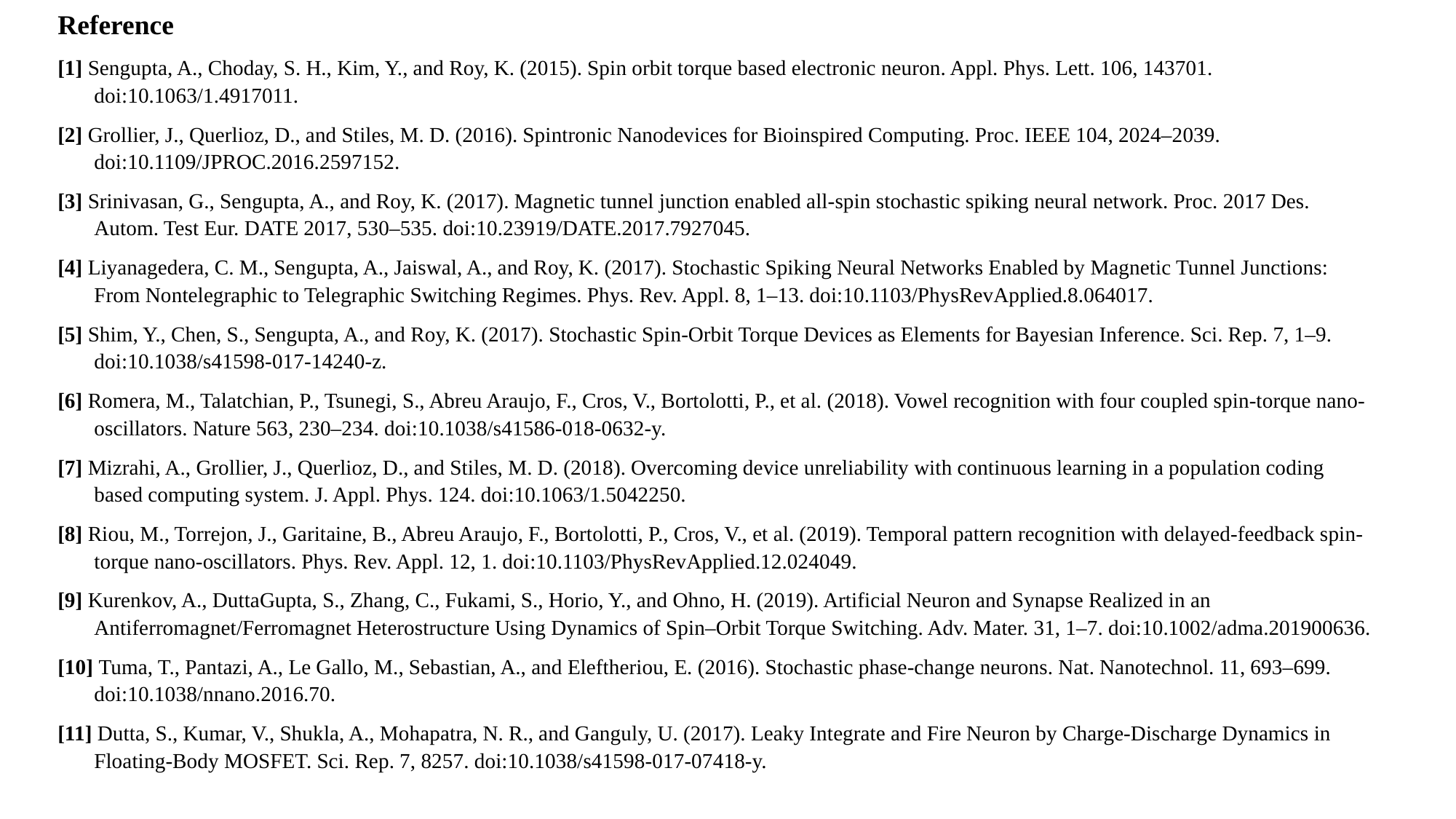

Reference
[1] Sengupta, A., Choday, S. H., Kim, Y., and Roy, K. (2015). Spin orbit torque based electronic neuron. Appl. Phys. Lett. 106, 143701. doi:10.1063/1.4917011.
[2] Grollier, J., Querlioz, D., and Stiles, M. D. (2016). Spintronic Nanodevices for Bioinspired Computing. Proc. IEEE 104, 2024–2039. doi:10.1109/JPROC.2016.2597152.
[3] Srinivasan, G., Sengupta, A., and Roy, K. (2017). Magnetic tunnel junction enabled all-spin stochastic spiking neural network. Proc. 2017 Des. Autom. Test Eur. DATE 2017, 530–535. doi:10.23919/DATE.2017.7927045.
[4] Liyanagedera, C. M., Sengupta, A., Jaiswal, A., and Roy, K. (2017). Stochastic Spiking Neural Networks Enabled by Magnetic Tunnel Junctions: From Nontelegraphic to Telegraphic Switching Regimes. Phys. Rev. Appl. 8, 1–13. doi:10.1103/PhysRevApplied.8.064017.
[5] Shim, Y., Chen, S., Sengupta, A., and Roy, K. (2017). Stochastic Spin-Orbit Torque Devices as Elements for Bayesian Inference. Sci. Rep. 7, 1–9. doi:10.1038/s41598-017-14240-z.
[6] Romera, M., Talatchian, P., Tsunegi, S., Abreu Araujo, F., Cros, V., Bortolotti, P., et al. (2018). Vowel recognition with four coupled spin-torque nano-oscillators. Nature 563, 230–234. doi:10.1038/s41586-018-0632-y.
[7] Mizrahi, A., Grollier, J., Querlioz, D., and Stiles, M. D. (2018). Overcoming device unreliability with continuous learning in a population coding based computing system. J. Appl. Phys. 124. doi:10.1063/1.5042250.
[8] Riou, M., Torrejon, J., Garitaine, B., Abreu Araujo, F., Bortolotti, P., Cros, V., et al. (2019). Temporal pattern recognition with delayed-feedback spin-torque nano-oscillators. Phys. Rev. Appl. 12, 1. doi:10.1103/PhysRevApplied.12.024049.
[9] Kurenkov, A., DuttaGupta, S., Zhang, C., Fukami, S., Horio, Y., and Ohno, H. (2019). Artificial Neuron and Synapse Realized in an Antiferromagnet/Ferromagnet Heterostructure Using Dynamics of Spin–Orbit Torque Switching. Adv. Mater. 31, 1–7. doi:10.1002/adma.201900636.
[10] Tuma, T., Pantazi, A., Le Gallo, M., Sebastian, A., and Eleftheriou, E. (2016). Stochastic phase-change neurons. Nat. Nanotechnol. 11, 693–699. doi:10.1038/nnano.2016.70.
[11] Dutta, S., Kumar, V., Shukla, A., Mohapatra, N. R., and Ganguly, U. (2017). Leaky Integrate and Fire Neuron by Charge-Discharge Dynamics in Floating-Body MOSFET. Sci. Rep. 7, 8257. doi:10.1038/s41598-017-07418-y.

## Slide 13
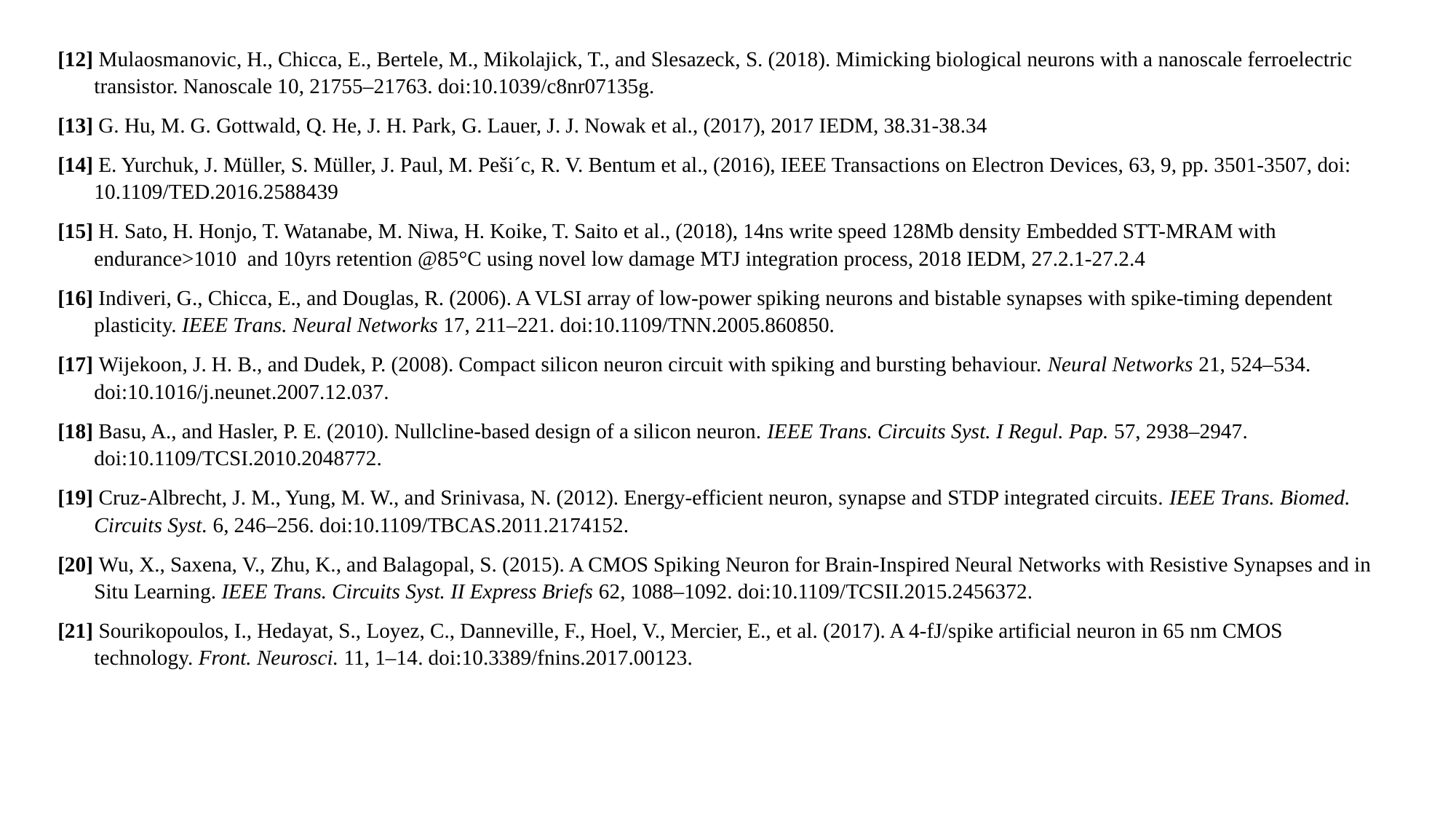

[12] Mulaosmanovic, H., Chicca, E., Bertele, M., Mikolajick, T., and Slesazeck, S. (2018). Mimicking biological neurons with a nanoscale ferroelectric transistor. Nanoscale 10, 21755–21763. doi:10.1039/c8nr07135g.
[13] G. Hu, M. G. Gottwald, Q. He, J. H. Park, G. Lauer, J. J. Nowak et al., (2017), 2017 IEDM, 38.31-38.34
[14] E. Yurchuk, J. Müller, S. Müller, J. Paul, M. Peši´c, R. V. Bentum et al., (2016), IEEE Transactions on Electron Devices, 63, 9, pp. 3501-3507, doi: 10.1109/TED.2016.2588439
[15] H. Sato, H. Honjo, T. Watanabe, M. Niwa, H. Koike, T. Saito et al., (2018), 14ns write speed 128Mb density Embedded STT-MRAM with endurance>1010 and 10yrs retention @85°C using novel low damage MTJ integration process, 2018 IEDM, 27.2.1-27.2.4
[16] Indiveri, G., Chicca, E., and Douglas, R. (2006). A VLSI array of low-power spiking neurons and bistable synapses with spike-timing dependent plasticity. IEEE Trans. Neural Networks 17, 211–221. doi:10.1109/TNN.2005.860850.
[17] Wijekoon, J. H. B., and Dudek, P. (2008). Compact silicon neuron circuit with spiking and bursting behaviour. Neural Networks 21, 524–534. doi:10.1016/j.neunet.2007.12.037.
[18] Basu, A., and Hasler, P. E. (2010). Nullcline-based design of a silicon neuron. IEEE Trans. Circuits Syst. I Regul. Pap. 57, 2938–2947. doi:10.1109/TCSI.2010.2048772.
[19] Cruz-Albrecht, J. M., Yung, M. W., and Srinivasa, N. (2012). Energy-efficient neuron, synapse and STDP integrated circuits. IEEE Trans. Biomed. Circuits Syst. 6, 246–256. doi:10.1109/TBCAS.2011.2174152.
[20] Wu, X., Saxena, V., Zhu, K., and Balagopal, S. (2015). A CMOS Spiking Neuron for Brain-Inspired Neural Networks with Resistive Synapses and in Situ Learning. IEEE Trans. Circuits Syst. II Express Briefs 62, 1088–1092. doi:10.1109/TCSII.2015.2456372.
[21] Sourikopoulos, I., Hedayat, S., Loyez, C., Danneville, F., Hoel, V., Mercier, E., et al. (2017). A 4-fJ/spike artificial neuron in 65 nm CMOS technology. Front. Neurosci. 11, 1–14. doi:10.3389/fnins.2017.00123.
